# Future scenarios for the SARS-CoV-2 epidemic in Switzerland: an age-structured model

**DOI:** 10.12688/f1000research.24497.1

**Published:** 2020-06-25

**Authors:** Janne Estill, Plamenna Venkova-Marchevska, Maroussia Roelens, Erol Orel, Alexander Temerev, Antoine Flahault, Olivia Keiser

**Affiliations:** 1 Institute of Global Health, University of Geneva, Geneva, GE, 1202, Switzerland; 2 Institute of Mathematical Statistics and Actuarial Science, University of Bern, Bern, BE, 3012, Switzerland

**Keywords:** COVID-19, SARS-CoV-2, mathematical model, Switzerland

## Abstract

The recent lifting of COVID-19 related restrictions in Switzerland causes uncertainty about the future of the epidemic. We developed a compartmental model for SARS-CoV-2 transmission in Switzerland and projected the course of the epidemic until the end of year 2020 under various scenarios. The model was age-structured with three categories: children (0-17), adults (18-64) and seniors (65- years). Lifting all restrictions according to the plans disclosed by the Swiss federal authorities by mid-May resulted in a rapid rebound in the epidemic, with the peak expected in July. Measures equivalent to at least 90% reduction in all contacts were able to eradicate the epidemic; a 56% reduction in contacts could keep the intensive care unit occupancy under the critical level and delay the next wave until October. In scenarios where strong contact reductions were only applied in selected age groups, the epidemic could not be suppressed, resulting in an increased risk of a rebound in July, and another stronger wave in September. Future interventions need to cover all age groups to keep the SARS-CoV-2 epidemic under control.

## Introduction

Switzerland has one of the highest incidences of documented SARS-CoV-2 infections per population, with large regional variability ^1, 2^. In response to the SARS-CoV-2 pandemic, the Swiss federal government implemented several restrictions, including closures of schools and non-essential shops and services and forbidding gatherings of more than five people ^3^. Many of these restrictions were lifted on May 11, 2020, causing uncertainty about the future of the epidemic. We have developed an age-structured mathematical model to estimate possible scenarios for Switzerland until December 2020, and to identify how different levels of contact reduction between different age groups influence SARS-CoV-2 transmission.

## Methods

### Model generation and fitting

We used a stochastic compartmental model. The population of Switzerland is divided into three age groups (children (0–17 years), adults (18–64 years) and seniors (≥65 years)) and 11 compartments that represent the epidemiological stage. Most parameters (disease progression; probability of different levels of symptoms) were directly adapted from a model for France ^4^. Relative contact frequencies between children, adults and seniors were retrieved from studies in Belgium, France, Germany and Italy ( http://www.socialcontactdata.org/) ^5, 6^. Initial conditions (starting date, initial number of exposed individuals), COVID-19 related mortality rates of adults and seniors, overall infectiousness and the relative efficacy of each preventive measure were estimated by calibrating the model results against the daily COVID-19 hospitalizations and deaths ^2, 7^. The code and a detailed description of the methodology, including all input parameters and their sources, is available at https://gitlab.com/igh-idmm-public/covid-19/modelling_jestill and is archived with Zenodo ^8^.

Before running the model for Switzerland, we fitted it to three cantons with epidemics that started at different times (Geneva, Ticino and Bern) to select informative priors for the uncertain parameters. We included the impact of the government-imposed restrictions. We modelled seven scenarios with different assumptions regarding the prevention after 11 May 2020: i) baseline scenario (no further preventive measures introduced); ii) eradication scenario (all contacts reduced by 90%); iii) epidemic control scenario (minimum contact reduction that can keep the number of ICU patients under the critical limit of 1200) ^9^; iv) same contact reduction among adults and seniors as in scenario 3 but without any restriction on children’s contacts; v) same contact reduction in contacts involving adults as in scenario 3, and half of that reduction for other contacts; vi) half of the contact reduction of scenario 3, but 95% contact reduction between seniors and other age groups; and vii) reintroduction of the full lockdown measures as between 20 March–26 April 2020 as soon as the daily new hospitalizations reach 40, until the daily new hospitalizations have remained below 10 for two weeks. We assumed in all scenarios that strong social distancing (restricting all larger gatherings of adults) would continue until 7 June, and “light” social distancing (awareness, hand hygiene) for the rest of the year. Relative reductions in contacts were calculated from this baseline assumption. Contacts are defined as all contact scenarios in which transmission is possible from a single infectious person, regardless of type or duration. We represented future interventions (including contact tracing, intensified screening, and wearing masks) by an overall reduction in either all contacts or contacts between specific age groups. We run the model deterministically until 11 May 2020 and stochastically thereafter, and present the means of 1000 simulations with 95% credible interval (CrI).

### Sensitivity analyses

We also conducted several sensitivity analyses to explore how the results depend on some key input assumptions. First, we shortened the latency period so that the serial interval decreased from 7.5 ^4^ to 4.8 ^10^. Second, we prolonged the infectiousness by adding a compartment of post-symptomatic infection to all infectious individuals except those who were hospitalized ^11^. Finally, we added a seasonal forcing, which is a potential but currently disputed factor ^12, 13^, multiplying the infectiousness by a factor ranging between 0.6 (mid-summer) and 1.0 (mid-winter).

## Results

### Modelling outcomes for each scenario

**Figure 1.**
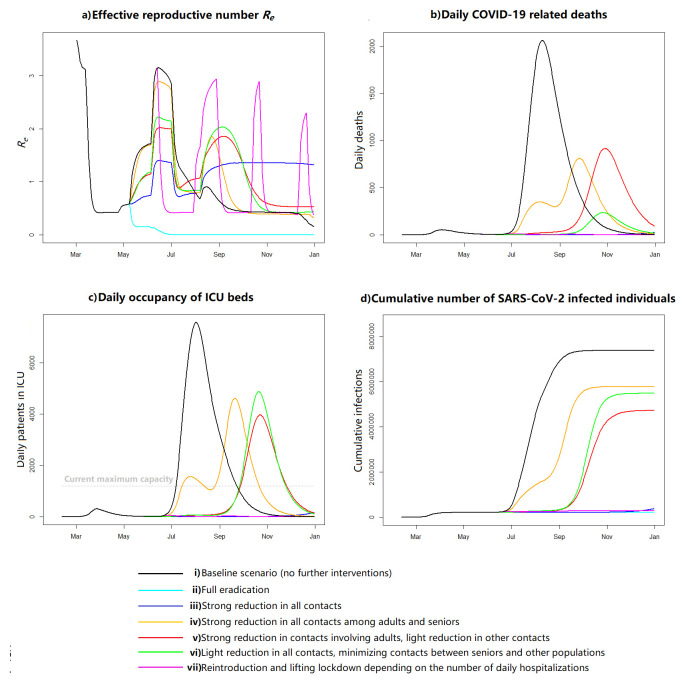
Results from different scenarios **a**) Reproductive number, **b**) daily COVID-19 related deaths, **c**) daily intensive care unit (ICU) bed occupancy, and **d**) cumulative infections in Switzerland from 11 February to 31 December 2020. “Strong reduction” refers to a 56% reduction of contacts (calibrated to prevent ICU overflow with 90% probability), “light reduction” is half of that, and “minimizing contacts” refers to 95% reduction of contacts.

Easing of restrictions may lead to a rapid increase in infections from late June, if the population relaxes social distancing and no effective tracing or testing efforts are implemented (baseline scenario, i). In this scenario, 83% of the Swiss population would become infected by the end of the year ( Figure 1). By restricting total contacts by 90% (eradication scenario, ii), we estimate a full eradication of the epidemic, resulting in no infectious individuals in Switzerland by 25 July. However, low overall immunity (<5%) leaves the population vulnerable to new outbreaks. A 56% reduction in all contacts would retain the occupancy of ICU beds below the current maximum capacity throughout the year (scenario iii). In this scenario, the effective reproductive number, *R _e_*, would stay around 1.4, decreasing to <1 only during summer holidays. In this scenario, a new wave would start in October and only 5.2% (95% CrI 3.4-7.6%) of the population would have been infected by the end of 2020.

Maintaining a minimum of 56% contact reduction for all age groups is essential. Scenario iv, where this reduction was applied only for contacts among adults and seniors, without restricting contacts involving children, would result in two peaks, in late July and late September, and a substantial ICU overflow and mortality from mid-July until mid-October. In this scenario, 64.9% (95% CrI 63.6-66.2%) of the population would have been infected by the end of 2020. When we reduced contacts involving adults by 56%, and contacts among children and seniors only by 28% (scenario v), we observed no peak in July but a larger peak in autumn, resulting in a similar disease burden (cumulative proportion of infected individuals 54.8%, 95% CrI 54.7-54.9%). A similar pattern in the epidemic, with the next strong peak in October, was also seen in scenario vi when we restricted all contacts by 28%, except those between seniors and other age groups by 95%. In this case the number of deaths was however four times lower and the cumulative proportion of infected higher (63.3%, 95% CrI 63.3-63.6%). All above-mentioned scenarios were sensitive to the parameterisation: for example, in the model calibration for the Canton of Geneva, restricting contacts among adults and seniors only resulted in a stronger peak during summer without the second peak, whereas the bimodal pattern was in turn seen in the two other scenarios.

In the last modelled scenario (vii), reintroducing and lifting the restrictions based on daily hospitalizations would result in four new lockdowns: 16 June–1 August 2020, 31 August–14 October 2020, 25 October–13 December 2020, and 24 December 2020–13 February 2021. The number of patients in ICU remained below 200 throughout. Overall 5.6% (95% CrI 4.8-6.6%) of the total population would have been infected by 31 December.

### Sensitivity analyses

The sensitivity analyses showed that the scenarios were sensitive to the duration of the latency period and infectiousness. Shorter serial interval made it easier to eradicate the epidemic or delay the second wave, whereas assuming post-symptomatic infectiousness meant that stronger contact reductions were needed to control the epidemic. Seasonal forcing also slowed down the epidemic: assuming a strong seasonality factor could delay the next wave to at least September.

## Discussion

Our study demonstrates that as long as the virus is present in a community with limited immunity, there is a risk of a rapid rebound of the epidemic if the restrictions are lifted and the people stop following social distancing and other protective behaviour. In the absence of seasonal forcing, the next wave could in theory occur in the summer. Efforts to control the epidemic, such as intensive testing, contact tracing, wearing of masks and hygiene measures, must have sufficient coverage to limit transmission among all age groups. No single intervention is enough. Reducing transmission uniformly may keep the epidemic suppressed only until late autumn. Restricting contacts between seniors and the rest of the population will prevent deaths, but is not sufficient to control the epidemic. A “start-stop” strategy, where the trends in COVID-19 related hospitalizations (or confirmed cases) trigger a new lockdown, is effective but not feasible: this would push the lockdown into the future, with only a few weeks of “normality” in between.

This study provides a broad range of future scenarios. In reality, the most severe scenarios are very unlikely: we can expect that the public health authorities will react and the people will adapt their behaviour if there is a new increase in cases. Our study has also several limitations. We focused on the average restriction of transmission-supporting contacts, without considering the practical implementation of specific interventions. The baseline contact patterns were based on literature data. They may however not fully reflect transmission patterns as the knowledge on transmission routes is still limited. Some contacts may be easier to track and control than others. Using a compartmental model, we could not differentiate between household and community transmission, or consider the effect of superspreaders.

An effective response to the COVID-19 pandemic needs several components: the spread of the infection must be kept as low as possible, the vulnerable population groups need additional protection, effective monitoring strategies must be in place, and the society must be ready to reintroduce additional measures if necessary. Only a combined prevention approach that targets all population groups can assure a sufficient control of the epidemic until a vaccine becomes available.

## Data availability

### Source data

We used the following data to parameterise our model:

Disease progression parameters (except mortality, infectiousness and relative contact frequency): https://www.medrxiv.org/content/10.1101/2020.04.13.20063933v1 Appendix S1.

Relative contact frequency: www.socialcontactdata.org (online tool https://lwillem.shinyapps.io/socrates_rshiny/, with data from studies “Belgium 2010”, “France 2015”, “Germany 2008” and “Italy 2008”).

Calibration for Switzerland and the Cantons of Bern and Ticino: www.corona-data.ch.

Calibration for the Canton of Geneva: https://www.ge.ch/document/covid-19-situation-epidemiologique-geneve.

### Extended data

**Model code available at:**https://gitlab.com/igh-idmm-public/covid-19/modelling_jestill/-/tree/master/.

**Archived code at time of publication:** http://www.doi.org/10.5281/zenodo.3888230

**License:**Creative Commons Attribution 4.0 International.
