# Supplementary material for: Future scenarios for the SARS-CoV-2 epidemic in Switzerland: an age-structured model
Source: F1000Res. 2021 Mar 11;9:646. Originally published 2020 Jun 25. [Version 2] doi: 10.12688/f1000research.24497.2 (PMC8477351; doi:10.12688/f1000research.24497.2)
Supplement: Peer review history

# Peer review: Reviewer response for version 2

**DOI:** 10.5256/f1000research.31154.r81309

**Published:** 2021-09-27

**Authors:** Andrei R. Akhmetzhanov

**Affiliations:** 1 National Taiwan University College of Public Health, Taipei, Taiwan

I am satisfied with response of the authors and I am happy to recommend it for approval.

Is the work clearly and accurately presented and does it cite the current literature?

Partly

If applicable, is the statistical analysis and its interpretation appropriate?

Yes

Are all the source data underlying the results available to ensure full reproducibility?

Yes

Is the study design appropriate and is the work technically sound?

Yes

Are the conclusions drawn adequately supported by the results?

Partly

Are sufficient details of methods and analysis provided to allow replication by others?

Partly

Reviewer Expertise:

Infectious disease modeling

I confirm that I have read this submission and believe that I have an appropriate level of expertise to confirm that it is of an acceptable scientific standard.

# Peer review: Reviewer response for version 2

**DOI:** 10.5256/f1000research.31154.r81310

**Published:** 2021-03-25

**Authors:** Subhas Khajanchi

**Affiliations:** 1 Department of Mathematics, Presidency University, Kolkata, India

The authors carefully revised the manuscript by including all the comments. I am in favor of indexing of the manuscript.

Is the work clearly and accurately presented and does it cite the current literature?

Partly

If applicable, is the statistical analysis and its interpretation appropriate?

Partly

Are all the source data underlying the results available to ensure full reproducibility?

Partly

Is the study design appropriate and is the work technically sound?

Yes

Are the conclusions drawn adequately supported by the results?

Partly

Are sufficient details of methods and analysis provided to allow replication by others?

Yes

Reviewer Expertise:

Infectious diseases, Ecological modeling, Tumor-immune interactions.

I confirm that I have read this submission and believe that I have an appropriate level of expertise to confirm that it is of an acceptable scientific standard.

# Peer review: Reviewer response for version 1

**DOI:** 10.5256/f1000research.27023.r74090

**Published:** 2020-11-16

**Authors:** Subhas Khajanchi

**Affiliations:** 1 Department of Mathematics, Presidency University, Kolkata, India

The COVID-19 pandemic has already spread throughout the world and the people are aware about the diseases and they are using precautions about the pandemic. But, still COVID-19 is spreading very quickly. Some countries like Spain, Switzerland, Australia, Serbia, China etc. started a second wave of COVID-19.

 To stop the spread of the diseases a vaccine is needed. But, in absence of the vaccine people must maintain the social distancing. In order to maintain the social distancing people must obey the modeling rule. The introduction needs to be improved by incorporating some recent references on the COVID-19 pandemic. To do so, I suggest some modeling work must be included in the references: Sarkar *et al.* (2020 ^1^), Khajanchi and Sarkar (2020 ^2^) and Samui *et al.* (2020 ^3^).

 In this context an important factor must be include in this study, that is, the impact of the effect of media. How the COVID-19 dynamics have been changed due to incorporation of the media-related awareness like use of face masks, non-pharmaceutical interventions, hand sanitization, etc. The authors must include the manuscript Khajanchi *et al.* (2020 ^4^) to study the effect of media.

 Is there any experimental data to validate the mathematical model? The authors at least describe the basic reproduction number R_0 and its impact on COVID-19 pandemic in India. The basic reproduction number R_0 is one of the most crucial quantities in infectious diseases, as R_0 measures how contagious a disease is. For R_0 < 1, the disease is expected to stop spreading, but for  R_0 = 1 an infected individual can infect on an average 1 person, that is, the spread of the disease is stable. The disease can spread and become epidemic if R_0 is be greater than 1. In this context the authors can read the manuscript Wang *et al.* (2002 ^5^).

 Some references contain errors and inconsistent formatting. It is difficult to give credit to research if even elementary aspects of the work are not error free. This should be corrected with care and love to detail.

 The manuscript is comprehensive, and I have enjoyed learning about the presented results. I find that the manuscript is written with poor English and the presentation is not good, and I am in principal in favor of indexing, although the following comments should nevertheless be accommodated in one minor revision.

Is the work clearly and accurately presented and does it cite the current literature?

Partly

If applicable, is the statistical analysis and its interpretation appropriate?

Partly

Are all the source data underlying the results available to ensure full reproducibility?

Partly

Is the study design appropriate and is the work technically sound?

Yes

Are the conclusions drawn adequately supported by the results?

Partly

Are sufficient details of methods and analysis provided to allow replication by others?

Yes

Reviewer Expertise:

Infectious diseases, Ecological modeling, Tumor-immune interactions.

I confirm that I have read this submission and believe that I have an appropriate level of expertise to confirm that it is of an acceptable scientific standard, however I have significant reservations, as outlined above.

## References

[1] : Modeling and forecasting the COVID-19 pandemic in India.*Chaos Solitons Fractals*.2020;139: 10.1016/j.chaos.2020.11004911004910.1016/j.chaos.2020.11004932834603PMC7321056

[2] : Forecasting the daily and cumulative number of cases for the COVID-19 pandemic in India.*Chaos*.2020;30(7) : 10.1063/5.001624007110110.1063/5.001624032752627PMC7585452

[3] : A mathematical model for COVID-19 transmission dynamics with a case study of India.*Chaos Solitons Fractals*.2020;140: 10.1016/j.chaos.2020.11017311017310.1016/j.chaos.2020.11017332834653PMC7405793

[4] : Dynamics of the COVID-19 pandemic in India. *arXiv*.2020;2005(06286) : Reference source

[5] : Mathematical analysis of the global dynamics of a model for HTLV-I infection and ATL progression. *Mathematical Biosciences*.2002;179(2) : 10.1016/S0025-5564(02)00103-7207-21710.1016/S0025-5564(02)00103-712208616

# Response

**Published:** 2020-12-01

**Authors:** Janne Estill

**Affiliations:** University of Geneva, Switzerland

**Dear Dr Khajanchi,**

**Many thanks for your constructive comments. Please see below our point-to-point response.**

**Sincerely**

**Janne Estill on behalf of all authors**

 The COVID-19 pandemic has already spread throughout the world and the people are aware about the diseases and they are using precautions about the pandemic. But, still COVID-19 is spreading very quickly. Some countries like Spain, Switzerland, Australia, Serbia, China etc. started a second wave of COVID-19.

 To stop the spread of the diseases a vaccine is needed. But, in absence of the vaccine people must maintain the social distancing. In order to maintain the social distancing people must obey the modeling rule. The introduction needs to be improved by incorporating some recent references on the COVID-19 pandemic. To do so, I suggest some modeling work must be included in the references: Sarkar *et al.* (2020 ^1^), Khajanchi and Sarkar (2020 ^2^) and Samui *et al.* (2020 ^3^).

**Authors’ response: Thank you for pointing this out. We have added a sentence into the Introduction highlighting that until large-scale vaccination, social distancing and other measures need to be kept in place. Since all references suggested by the reviewer refer to India, which is not the focus of this study, we have taken the liberty to instead give references more related to the epidemic in Switzerland and Europe.**

**“ *A vaccine is likely to be available in the course of the year 2021, but until then, strict social distancing and reduction of contacts together with measures such as testing and tracing are needed to control the epidemic and prevent the health system from a collapse.”***

 In this context an important factor must be include in this study, that is, the impact of the effect of media. How the COVID-19 dynamics have been changed due to incorporation of the media-related awareness like use of face masks, non-pharmaceutical interventions, hand sanitization, etc. The authors must include the manuscript Khajanchi *et al.* (2020 ^4^) to study the effect of media.

**Authors’ response: We agree that the media plays an important role in the network of determinants that influence transmission dynamics. As mentioned in the second paragraph of the Methods, we do not aim to model explicit interventions from the summer onwards, but only use implicit contact reduction levels between children, adults and seniors. We have added the following statement into the Methods (second paragraph):**

***“The future reduction contains both government-ordered measures (such as the mandatory use of masks, new closures of services or locations), as well as indirect influence on behaviour through e.g. efficient communication by the health authorities as well as the mass and social media.*“**

 Is there any experimental data to validate the mathematical model? The authors at least describe the basic reproduction number R_0 and its impact on COVID-19 pandemic in India. The basic reproduction number R_0 is one of the most crucial quantities in infectious diseases, as R_0 measures how contagious a disease is. For R_0 < 1, the disease is expected to stop spreading, but for  R_0 = 1 an infected individual can infect on an average 1 person, that is, the spread of the disease is stable. The disease can spread and become epidemic if R_0 is be greater than 1. In this context the authors can read the manuscript Wang *et al.* (2002 ^5^).

**Authors’ response: The model has been calibrated with the observed hospitalization and death data, as explained in the first paragraph of the Methods section. We focus in our paper on the effective reproduction number (Re; see Figure 1a for an estimate of Re over time in all scenarios) instead of the basic reproductive number (R0). These are by definition equivalent in a completely susceptible population, while the Re decreases as the proportion of the population becomes immune. We prefer to use Re instead of R0 since this is used also by e.g. the Swiss COVID-19 Task Force situation report ( https://ncs-tf.ch/en/situation-report), allowing a comparison between different modelling studies for Switzerland. We trust that the concept of effective reproduction number is known to the readers and will not require further clarification.****

 Some references contain errors and inconsistent formatting. It is difficult to give credit to research if even elementary aspects of the work are not error free. This should be corrected with care and love to detail.

**Authors’ response: We have carefully checked all references and found no errors in the links. We trust the editorial office will point out any remaining issues with the references.**

 The manuscript is comprehensive, and I have enjoyed learning about the presented results. I find that the manuscript is written with poor English and the presentation is not good, and I am in principal in favor of indexing, although the following comments should nevertheless be accommodated in one minor revision.

**Authors’ response: The manuscript has been edited for grammar and style by a native English speaker. We are willing to improve the presentation in our manuscript by further editing, but it is difficult to address this comment without specific details.**

# Peer review: Reviewer response for version 1

**DOI:** 10.5256/f1000research.27023.r68276

**Published:** 2020-08-10

**Authors:** Andrei R. Akhmetzhanov

**Affiliations:** 1 National Taiwan University College of Public Health, Taipei, Taiwan

The paper of Estill et al. reports on possible future scenarios for SARS-CoV-2 epidemic in Switzerland. The results look plausible and may have important implications for a policy-making process. However, I think the study requires a more detailed sensitivity analysis and more flexibility in the proposed scenarios. In my opinion, the adopted values of the parameters (90% of reduction in contacts, 56% reduction, etc.) could be more variable, and additional analysis could help to determine their most efficient values. I also think that the Discussion section could propose a better overview of the problem and give comparison with other studies. Some other remarks of mine are below.

- Figure 1b vs 1c: I would have thought that the deaths counts should be lagged by approx. two weeks compared to ICU bed occupation. However, the black curve in 1b peaks visually at the same time as in 1c. Could the authors check this more carefully and confirm that there is some lag? 
- Figure 1d vs 1c: What confuses me more is that there is no visually distinct time lag between reported cases and deaths.
- Figure 1: what are the Credible intervals for each trajectory?
- Compartmental model: it would be nice to have at least a brief description of the adopted model. For example, whether it includes asymptomatic carriers, hospitalized/quarantined people. It would be also good to know if the transition times follow exponential distribution or are described by gamma distribution. The authors could elaborate more than simply saying that all parameters are directly adopted from Ref.4.
- I believe that the considered scenarios may represent some minimalistic set, but it would be also nice to have a more detailed sensitivity analysis. For example, the reduction of contacts in Japan was set to 80% during the first wave, and it was proven to be sufficient. The authors of the present study consider the reduction at 90%, which may be more difficult to implement. Hence, I may have a question: how much should we actually reduce the contact rate?
- I may have a similar question regarding the seasonal forcing and choice of 7 June for lighting up the social restrictions. What would be a reasonable choice of such date? Is 7 June chosen optimally?
- I am a bit unsure if students (in 20s) and workforce subpopulation (in 30s-40s) can be attributed to the same age group. In my opinion, they have a quite distinct contact pattern. 

Is the work clearly and accurately presented and does it cite the current literature?

Partly

If applicable, is the statistical analysis and its interpretation appropriate?

Yes

Are all the source data underlying the results available to ensure full reproducibility?

Yes

Is the study design appropriate and is the work technically sound?

Yes

Are the conclusions drawn adequately supported by the results?

Partly

Are sufficient details of methods and analysis provided to allow replication by others?

Partly

Reviewer Expertise:

Infectious disease modeling

I confirm that I have read this submission and believe that I have an appropriate level of expertise to confirm that it is of an acceptable scientific standard, however I have significant reservations, as outlined above.

# Response

**Published:** 2020-12-01

**Authors:** Janne Estill

**Affiliations:** University of Geneva, Switzerland

**Dear Dr Akhmetzhanov,**

**Thank you for your constructive comments. Please see our point-to-point response below.**

**Sincerely**

**Janne Estill on behalf of all authors**

 The paper of Estill et al. reports on possible future scenarios for SARS-CoV-2 epidemic in Switzerland. The results look plausible and may have important implications for a policy-making process. However, I think the study requires a more detailed sensitivity analysis and more flexibility in the proposed scenarios. In my opinion, the adopted values of the parameters (90% of reduction in contacts, 56% reduction, etc.) could be more variable, and additional analysis could help to determine their most efficient values. I also think that the Discussion section could propose a better overview of the problem and give comparison with other studies. Some other remarks of mine are below.

**Authors’ response: Thank you. The contact reduction in scenario iii) was not predefined at 56%: the value was selected after a calibration process as the minimum that allowed to keep the ICU occupancy under the critical limit (1200 beds) throughout the year in at least 90% of the simulations. After rerunning the analysis, we have corrected the value to 54%, which produces more stable results fulfilling the condition. We agree that this was imprecisely expressed and have revised the Methods to clarify that the reduction in itself is a result:**

***“The reduction levels for scenarios ii) and iii) were selected in a manual calibration process, increasing the reduction in increments of 1% until the target condition was met.”***

**The 90% reduction was pre-defined in our original analysis. We have now replaced the fixed 90% threshold also with a target-based scenario, calibrated to reaching a complete eradication (daily cases suppressed persistently to zero) by the beginning of July in at least 90% of the simulations. As a result, we have replaced the 90% overall reduction with a 76% reduction, which was the minimum level to fulfil this condition. Our revised description of Scenario ii) in the Methods (first paragraph) reads now as follows:**

***“ii) eradication scenario (minimum contact reduction to bring the daily new infections to zero by the end of July with 90% probability)”***

**The contact reductions in the remaining analyses were derived from the value 56% (either the same value, or half i.e. 28%), with the exception of the 95% reduction among contacts of seniors in Scenario vi) which was pre-defined (aiming to represent almost-isolation measures). We have decided to keep the same approach, now using the value of 54% instead of 56% (and 27% instead of 54%).**

 Figure 1b vs 1c: I would have thought that the deaths counts should be lagged by approx. two weeks compared to ICU bed occupation. However, the black curve in 1b peaks visually at the same time as in 1c. Could the authors check this more carefully and confirm that there is some lag? 

**Authors’ response: Because of the narrow graph, it is difficult to compare the peak values across panels. For example, in the baseline scenario (black curves), ICU occupancy peaked on 1st August, deaths on 9th August. The difference is 8 days, which is less than two weeks; however, the peak in ICU occupancy does not correspond to the peak in new ICU entries.  **  

 Figure 1d vs 1c: What confuses me more is that there is no visually distinct time lag between reported cases and deaths.

**Authors’ response: New infections in the baseline scenario (black curves) peaked on 23th July, which is 17 days before the peak in deaths (9th August). It is understandably difficult to see the peak from the cumulative curve in Fig. 1d. However, we find the cumulative infections more informative than daily infections, because the reported cases do not correspond to true infections (but instead, the cumulative cases can easily be compared with seroprevalence estimates once such become available). We have added a number of new supplementary graphs (including also the daily infections), which will help the interpretability.**

****

 Figure 1: what are the Credible intervals for each trajectory?

**Authors’ response: Because of the large number of graphs in each panel, including the credible intervals would probably make the graph unreadable. We have therefore instead included a set of separate supplementary online graphs (see document “covid_model_supplementary_figures.pdf” in the Gitlab repository https://gitlab.com/igh-idmm-public/covid-19/modelling_jestill) with the 95% credible intervals and all 1000 simulations into the appendix.**

****

 Compartmental model: it would be nice to have at least a brief description of the adopted model. For example, whether it includes asymptomatic carriers, hospitalized/quarantined people. It would be also good to know if the transition times follow exponential distribution or are described by gamma distribution. The authors could elaborate more than simply saying that all parameters are directly adopted from Ref.4.

**Authors’ response: Our aim was to keep this research paper in a form as a short report. All parameters and a complete description with a graphical representation of the model structure are available from the dedicated Gitlab repository. This is declared at the end of the first paragraph of the Methods.**

 I believe that the considered scenarios may represent some minimalistic set, but it would be also nice to have a more detailed sensitivity analysis. For example, the reduction of contacts in Japan was set to 80% during the first wave, and it was proven to be sufficient. The authors of the present study consider the reduction at 90%, which may be more difficult to implement. Hence, I may have a question: how much should we actually reduce the contact rate?

**Authors’ response**: **Please see our response to the first question. The scenario iii) (before 56%, now 54% reduction), and after our revision also scenario ii) (before 90%, now 76% reduction) are set to fulfil given targets. To answer the question, in order to keep the epidemic under control at least 54% reduction throughout the year is needed. In order to completely eradicate the epidemic during the summer, at least 76% reduction (together with a strict control to stop imported infections) would have been needed.**

**We have reformulated the statement in the first paragraph of the Discussion to answer these questions more directly:**

***“Reducing contacts between all people uniformly by at least 54% is needed to control the epidemic until the end of the year, but even in this case the restrictions would need to be tightened during the winter to avoid a rebound in January.”***

**In addition, we have added a short paragraph to the Discussion to put these estimates into context with the true evolvement of the epidemic in the summer and autumn:**

***“The second wave of COVID-19 hit Switzerland severely during October-November 2020. A comparison of the true course of the epidemic and the modelled scenarios shows that Switzerland achieved a relatively high reduction in contact frequencies during the summer and autumn 2020 with measures such as contact tracing, social distancing and mask obligation in public transport. These measures were however not sufficient to control the epidemic. Our findings are however promising in the sense that the overall contact reductions have likely been between 27 and 54% (scenarios iii to vi), giving hope that the new measures introduced by the Swiss Federal Council on 19 and 29 October 2020 (in combination with more stringent measures by many cantons) can, if sustained throughout the winter season, keep the epidemic under control.* *”***

 I may have a similar question regarding the seasonal forcing and choice of 7 June for lighting up the social restrictions. What would be a reasonable choice of such date? Is 7 June chosen optimally?

**Authors’ response: The Swiss government announced beforehand 8 June as the date of lifting most restrictions, such as the reopening of many leisure and tourism related destinations and easing the limits of public gatherings and events. We expected therefore that the social contacts would increase at this time. The opening was later changed to be two days earlier, 6 June. We have added the following statement:**

***“(declared beforehand by the authorities as the end date of most restrictions related to leisure activities)* *”***

 I am a bit unsure if students (in 20s) and workforce subpopulation (in 30s-40s) can be attributed to the same age group. In my opinion, they have a quite distinct contact pattern. 

**Authors’ response: We agree that this is a limitation, particularly in terms of leisure activities where the younger adults can be expected to have more contacts. However we do not believe that the work- and study-related contacts would differ substantially. More than half of the Swiss youth make a vocational education with apprenticeship after compulsory school and enter the workforce, whereas university students (who were affected by all universities switching to online teaching during the lockdown) are more likely to attend jobs where home office is feasible (also de facto mandatory in most cases during the first lockdown). Therefore we have a reason to believe that the differences in contact patterns within the “adult” group are less substantial than the contact patterns compared with children or seniors.**

**We have added the following statement to highlight the limitations:**

***“The division to three age groups cannot catch all heterogeneity in contact patterns between (for example, the more diverse social contacts of students and other young adults versus middle-aged with families; or the differences between healthy seniors slightly above the age of 65 versus more elderly nursing home residents with restricted mobility). However, we believe that the most essential contact differences are covered by the three age groups.* *”***
